# Monitoring of Heart Rate and Activity Using Telemetry Allows Grading of Experimental Procedures Used in Neuroscientific Rat Models

**DOI:** 10.3389/fnins.2020.587760

**Published:** 2020-12-17

**Authors:** Laura Wassermann, Simeon O. A. Helgers, Ann-Kristin Riedesel, Steven R. Talbot, André Bleich, Kerstin Schwabe, Christine Häger

**Affiliations:** ^1^Institute for Laboratory Animal Science, Hannover Medical School, Hanover, Germany; ^2^Department of Neurosurgery, Hannover Medical School, Hanover, Germany

**Keywords:** intracranial surgery, behavior, injection, *k*-means and SVM algorithms, welfare, telemetry

## Abstract

In animal experimentation, welfare and severity assessments of all procedures applied to animals are necessary to meet legal and ethical requirements, as well as public interests. So far, the methods suggested for this purpose are time consuming and personnel intensive. Also, evidence-based biostatistical methods for this purpose are still rare. We here tested whether the classification of heart rate (HR) and activity (Act) data monitored by telemetry in the home cage by unsupervised *k*-means-based class-labeling and subsequent Support Vector Machine (SVM) analysis allows severity assessment and grading of experimental procedures of different domains, including surgery, injection, behavioral testing, and routine handling for maintenance. Telemetric devices were subcutaneously implanted in young adult male Crl:CD(SD) and BDIX/UImHanZtm rats. After recovery, rats were randomly subjected to different experimental procedures, i.e., handling and cage change as routine maintenance, Rat Grimace Scale, burrowing, and social interaction for welfare assessment, as well as repeated subcutaneous injections. Thereafter, rats were either intracranially implanted with electrodes or injected with tumor cells. Directly after each procedure, HR and Act were monitored by telemetry in the home cage for 4 h. Application of *k*-means and SVM algorithms on the obtained data sets from baseline (as no stress), cage change (exploratory Act), and intracranial surgery (as burden) measurements computed three classes described as low HR/low Act, high HR/high Act, and high HR/low Act, respectively. Validation of the SVM model by entering data from all procedures confirmed the allocation to the high HR/low Act class (burden) after surgery, which lasted longer after subcutaneous transmitter implantation than after intracranial surgery. The majority of data points from repeated injections, behavioral testing, and maintenance handling were allocated to the low HR/low Act and high HR/high Act classes. Overall, the SVM model based on HR and Act data monitored in home cage after procedures may be useful for the classification and grading of experimental procedures of different domains.

## Introduction

In animal research, effective management of pain, discomfort, and stress is necessary not only for ethical and legal considerations but also to achieve high-quality science free from confounding pathophysiological factors. In many countries, both prospective and actual severity assessments of animal experiments are legally required. This provides the basis for an ethical evaluation and the conclusion if an animal experiment is justified to be conducted. In the field of neuroscience, complex experimental settings are often used, involving neurosurgical intervention, repeated injections, and behavioral testing (Wu et al., 2018; Al-Afif et al., 2019; Elle et al., 2020). Complicating this issue, the burden an animal experiences during procedures not only seldom consists of only one dimension, such as pain after surgery, but also includes other dimensions, such as emotional components in potentially fear-provoking foreign settings. Therefore, robust and meaningful severity assessment schemes addressing both pain and emotional components are necessary (Bleich and Tolba, 2017).

So far, clinical scoring and body weight have been mostly used to assess animals’ well-being within an experiment, but other procedures that take into account species-specific behaviors, such as burrowing or facial expression measured with the Rat Grimace Scale, are increasingly regarded as important (Keubler et al., 2018). Most of these methods require interactions with experimenters, which may be problematic in flight animals since these hide their reduced general state (Stasiak et al., 2003). Instead, telemetry allows home cage-based contactless and observer-unbiased monitoring of physiological parameters, such as heart rate (HR) and activity (Act), and allows, therefore, evidence-based severity assessment (Kramer et al., 1993; Arras et al., 2007; Cesarovic et al., 2011; Seiffert et al., 2019). A further benefit of telemetry is the high temporal resolution to detect short-term deviations of parameters (Kumstel et al., 2020).

Elevated HR has been described not only as physiological indicator for postoperative pain in various species (Kramer et al., 1993; Arras et al., 2007; National Academy of Sciences US, 2009; Cesarovic et al., 2011; Kumstel et al., 2020) but also as response to restraint (Sharp et al., 2002; Meijer et al., 2006; Chen et al., 2009), as response to handling (Kramer et al., 1993; Baturaite et al., 2005; Van Bogaert et al., 2006), or as response to injections (Meijer et al., 2006; Van Bogaert et al., 2006), indicating a stress-like reaction. However, elevated HR is also a physiological reaction to enhanced Act, e.g., during exploration of a novel environment (Van Den Buuse et al., 2001) or after cage change (Sharp et al., 2002; Van Bogaert et al., 2006; Meller et al., 2011).

Another parameter that has been reported to be altered in burdensome situations is Act. A reduction of this parameter has been described after potentially painful transmitter implantation (Greene et al., 2007; Bourque et al., 2010; Cesarovic et al., 2011; Kumstel et al., 2020). Act measured as voluntary wheel-running in mice was also reduced after repeated restraint stress (Häger et al., 2018). However, an increase of Act is found during the exploration of novel and unfamiliar situations (Galani et al., 2001), after cage change (Van Bogaert et al., 2006), or as a response to handling (Clement et al., 1989).

Simply thresholding HR and Act for severity ranking of procedures, however, would not acknowledge the complex interaction between both parameters. It would not take into account that enhanced HR with normal or reduced movement may indicate “burden” of the animal, whereas enhanced HR, together with enhanced Act, would be normal physiological effects of normal Act. Because of the intense interaction of HR and Act, it is difficult to use these parameters individually.

More recently, distinct readout parameters have been assessed and combined by biostatistical methods for severity classification. Receiver operating characteristic curves and principal component analysis have been used to assess the performance of parameters in severity assessment (Möller et al., 2018; Abdelrahman et al., 2019). Furthermore, clustering of wheel-running and body weight data using a *k*-means algorithm enabled the grading of severity levels during acute colitis, as well as under restraint stress (Häger et al., 2018). Nevertheless, experimental procedures of different domains, e.g., invasive surgery and injection, as well as non-invasive handling for maintenance and behavioral testing, have not been compared and thereby graded within one setting, yet.

We here telemetrically measured HR and Act of eight adult male Crl:CD(SD) (SD) and seven adult male BDIX/UImHanZtm (BDIX) rats for after application of different experimental procedures routinely used in our laboratory. These comprise not only intracranial implantation of electrodes for stimulation and recording of neuronal Act (Elle et al., 2020) and intracranial tumor cell injection for testing anti-tumor effects of local therapeutics (Wu et al., 2018) but also repeated subcutaneous injection, behavioral procedures for severity assessment (e.g., burrowing, grimace scaling, social interaction), and routine animal maintenance (handling and cage change). Using HR and Act data recorded for 4 h after each procedure in the home cage, we developed and verified a Support Vector Machine (SVM) model to classify and grade the impact of these procedures in the context of severity. As focus was on the short-term consequences of different procedures, only measures of the first 4 h after each procedure were used for our algorithm.

## Materials and Methods

### Ethics Statement

Experiments were approved by the Lower Saxony State Office for Consumer Protection and Food Safety (LAVES, license 18/2837). All efforts were made to minimize the pain or discomfort for the animals. All animal experiments followed the ARRIVE guidelines and were carried out in accordance with the EU Directive 2010/63/EU for animal experiments, including approval by local authorities and an animal ethics committee.

### Animals

The data of 15 male adult rats (8 weeks, 180 g) of two different strains were used in this study, i.e., eight adult male SD obtained from Charles River (Charles River Laboratories, Germany) and seven adult male BDIX rats obtained from the Central Animal Facility (Hannover Medical School, Hannover, Germany). In order to meet the criterion of reduction of laboratory animals, this study was embedded in ongoing research projects. The SD rat strain is used as a standard in our laboratories for electrode implantation for neuronal recording and stimulation. For intracranial tumor model, the BDIX rat strain is used, because it is congenic to the BT4Ca cells and can therefore be used without immunosuppression (Wu et al., 2018). The rats’ health status was monitored by a sentinel program following the FELASA recommendations. Health monitoring included daily clinical scoring and weight measures.

The rats were single housed in Type III open cages with wood chip bedding material (Espentiereinstreu AB P3; AsBe-wood GmbH, Gransee, Germany) in a ventilated cabinet (ScanTainer^®^; Scanbur, Denmark) under controlled environmental conditions (22 ± 2°C and 55 ± 10% humidity) with a 14 h light–10 h dark cycle (06:00–20:00 lights on).

The animals were fed with pelleted diet (1,324 TPF from Altromin Spezialfutter GmbH&Co., KG, Lage, Germany) and tap water *ad libitum*. Cages were changed once a week. Access to the animal room was restricted to experimenters.

### Study Design

**FIGURE 1:**
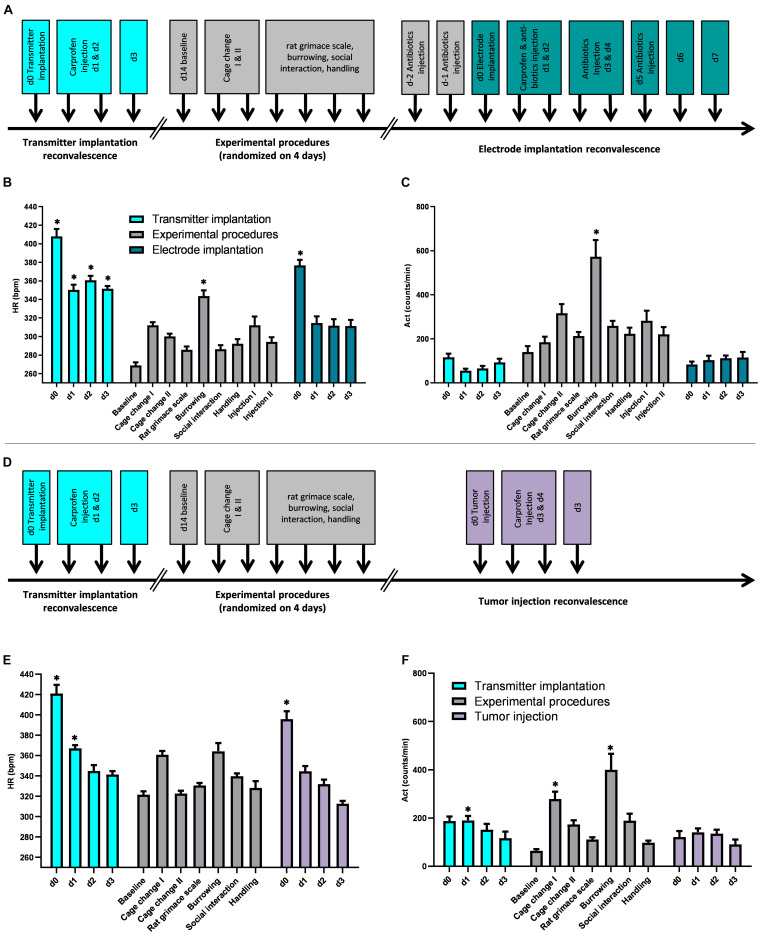
Telemetric HR and Act data. Timeline of experimental setup, mean heart rate (HR; bpm), and summed activity (Act; counts/min) for 4 h after indicated procedures of SD rats **(A–C)** and BDIX rats **(D–F)**. Data showed increased HR after surgery without increased Act and increased HR and Act after some experimental procedures. Colors indicate different procedures (turquoise—transmitter implantation, gray—experimental procedures, dark green—electrode implantation, purple—tumor injection). Significant differences are indicated by asterisk (*Friedman test, followed by Dunn’s multiple comparisons *post hoc* test against baseline; *P* < 0.05). Data are shown as mean + SEM.

After acclimatization in the animal facility, all rats were implanted with a telemetric device subcutaneously for continuous measurements of Act and HR. Following 2 weeks of recovery, rats were randomly subjected to different experimental procedures for maintenance (handling and cage change) and procedures for severity assessment (Rat Grimace Scale, burrowing, social interaction) with at least 1 day in between procedures. Four weeks after the implantation of the telemetric device, the SD rats were stereotactically implanted with two intracranial electrodes, whereas the BDIX rats received a stereotactic injection of BT4Ca cells for intracranial glioblastoma formation (Figure 1). Perioperatively, rats received subcutaneous injections of analgesics (Carprofen, RIMADYL^®^; Pfizer GmbH, Berlin, Germany), and rats with electrode implantation were additionally subcutaneously injected with antibiotics (Marbofloxacin, Marbocyl^®^ FD 1%; Vétoquinol GmbH, Ismaning, Germany).

At all days of interest, telemetric data of HR and Act were measured by Ponemah 6.41 (PhysioTel Telemetry System DSI; St Paul, MN, United States).

### Surgery

For general anesthesia, chloral hydrate (Sigma-Aldrich Chemie GmbH, Steinheim, Germany; initially 360 mg/10 ml/kg intraperitoneally, reinjection: up to 1/3 of the initial dose) was used. Additionally, 2% xylocaine (AstraZeneca GmbH, Wedel, Germany) was administered as a local anesthetic. For analgesia, rats received Carprofen (5 mg/kg intraoperatively, 2.5 mg/kg for 2 days postoperatively) after each surgery. For SVM analysis, the day of surgery and the following 3 postoperative days were used.

### Telemetric Transmitter Implantation

The telemetric device (ETA F10; PhysioTel Telemetry System DSI; St Paul, MN, United States) was subcutaneously implanted. A 2 cm long incision was made at the left abdominal side laterally to the spine, caudal to the costal arch, and a subcutaneous pocket was formed, where the transmitter was placed afterward. To build a derivation after Einthoven no. II, the electrodes were subcutaneously tunneled (the negative electrode was placed at the right pectoral region and the positive electrode at the end of the costal arch approximately 1 cm left laterally to the xiphoid). Both electrodes were fixed at their location by a single subcutaneous suture, and thereafter, the incisions were closed by sutures and suture clips. Although the transmitter device would allow recording of temperature, this data was not used for further analysis, because the subcutaneous temperature sensor did not give reliable measures.

### Stereotactic Surgery for Intracranial Electrodes Implantation and Injection of Tumor Cells

For stereotactic surgery, rats were placed in a stereotaxic frame in a flat skull position. After incising the skin and preparing the surgical site, Bregma was defined and used as a reference for intracranial targeting.

The SD rats were bilaterally implanted with platinum–iridium electrodes into the subthalamic nucleus through burr holes (anteroposterior: -3.8 mm, mediolateral: ± 2.5 mm, dorsoventral: -8.0 mm). The electrodes were fixed to the skull by dental acrylic cement (Paladur^®^; Heraeus Kulzer GmbH, Hanau, Germany) and four anchor screws. Rats received a daily Marbofloxacin injection (6.6 mg/kg subcutaneously) for 8 days, starting at 2 days preoperatively.

BDIX rats received an injection of a cell suspension containing malignant neuroectodermal cells (10^4^ BT4Ca glioma cells in 3 μl PBS) through a burr hole into the right frontal cortex (anteroposterior: +2.6 mm, mediolateral: +2.5 mm, dorsoventral: -2.8 mm). All incisions were closed by suture clips.

BDIX rats were euthanized and transcardially perfused when reaching the endpoint criterion of deteriorated clinical score and weight loss. SD rats were decapitated under CO_2_ anesthesia, and the brain was removed for molecular analysis.

### Experimental Procedures

#### Maintenance

*Handling* included grasping the rats with both hands around the chest, as used, e.g., for weight measurements. For *cage change*, rats were placed into a new cage with fresh bedding once a week.

#### Procedures for Severity Assessment

To evaluate the impact of the procedure itself, rats naïve to all procedures were used. For the *Rat Grimace Scale*, the rats were placed in cubical boxes (21 × 10.5 × 9 cm), set into the spotlight, and filmed for 5 min. To assess species-specific *burrowing* behavior, animals were placed in an empty Type IV cage for 30 min. Thereafter, the burrowing tube (320 × 100 mm plastic tube filled with 2.5 kg of gravel) was placed into the cage. One hour later, the amount of gravel burrowed was weighed. For *social interaction*, an age-matched unknown partner was introduced to the experimental rats in an empty Type V cage for 5 min. For standardization, all procedures were performed at the same time of the day.

#### Injections

To evaluate the impact of subcutaneous injections, the first two injections of antibiotics (i.e., injection 2 days before intracranial surgery) were used.

### Telemetric Data Analysis

The telemetric data of HR and Act were acquired by Ponemah 6.41 at days of interest. For evaluation, the telemetric data were split into three major groups: subcutaneous implantation of the telemetric device (displayed turquoise in figures), experimental procedures (gray; including procedures for maintenance, for severity assessment, and for subcutaneous injections), and intracranial surgery [which differs in both strains: SD rats—electrode implantation (dark green) and BDIX rats—tumor cell injection (purple)], see also experimental design in Figure 1.

Telemetric data measured were analyzed for 4 h after each experimental procedure. The HR was taken as mean in beats per minute (bpm), and the Act was taken as a sum of counts/min per indicated time interval. Recordings 2 weeks after transmitter implantation were taken as baseline measurements, as the animals are assumed to be fully recovered from the first surgery.

**FIGURE 2:**
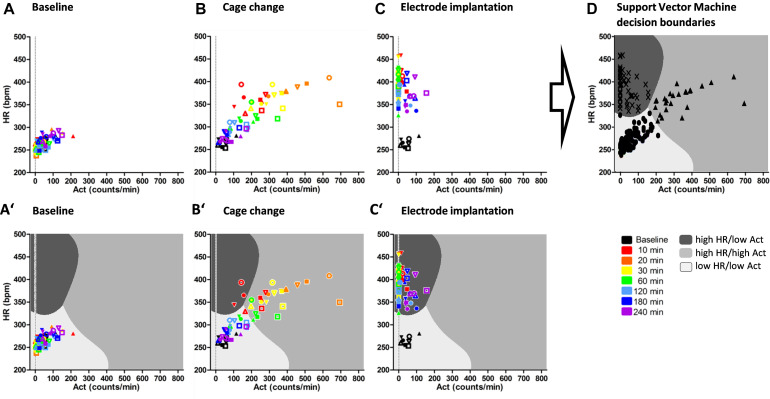
Development of classification model in SD rats. Heart rate (HR; bpm) and activity (Act; counts/min) until 4 h after experimental procedures of baseline as no stress **(A)**, cage change as stress-like response inducing procedure **(B)**, and electrode implantation as distress **(C)**. **(A–C)** served as training data set for development of Support Vector Machine (SVM) decision boundaries map **(D)** with blinded training data set (black symbols). SVM identified three classes: dark gray: high HR/low Act, midgray: high HR/high Act, light gray: low HR/low Act. Overlay of training data and decision boundaries **(A’–C’)**. HR is shown as mean, and Act is shown as sum over 10 min starting at the indicated time points (color-coded). Symbols indicate different animals.

**FIGURE 3:**
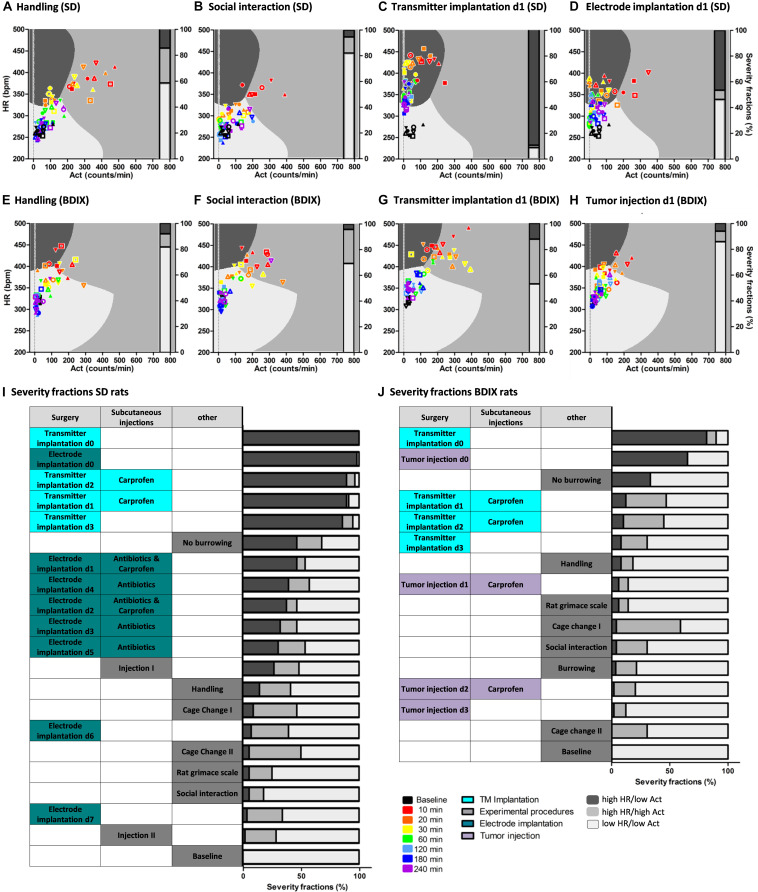
Evaluation of SVM as tool to classify severity. Exemplarily validation data plotted on SVM decision boundaries **(A–H)**: handling [**(A)** SD rats, **(E)** BDIX rats], social interaction [**(B)** SD rats, **(F)** BDIX rats], transmitter implantation d1 [**(C)** SD rats, **(G)** BDIX rats], electrode implantation d1 in SD rats **(D)**, and tumor injection in BDIX rats **(H)**. HR (HR; bpm) is shown as mean, and activity (Act; counts/min) is shown as sum over 10 min starting at the indicated time points (color coded) after the experimental procedure. Symbols indicate different individual animals. Three classes were identified by SVM: high HR/low Act (dark gray), high HR/high Act (midgray), low HR/low Act (light gray). Bars indicate fractions of data points allocated to different classes. Ranking of all experimental procedures according to the fraction of data points allocated to the high HR/low Act class [**(I)** SD rats, **(J)** BDIX rats].

Within the 4 h period after each experimental procedure, the HR was plotted against the Act for the following time bins (colors coded in Figures 2, 3): 0–10 (red), 10–20 (orange), 20–30 (yellow), 30–60 (green; downscale to 10 min), mean of 60–120 (light blue; downscale to 10 min), mean of 120–180 (dark blue; downscale to 10 min), and mean of 180–240 (purple; downscale to 10 min).

### Statistics

#### Training of the *k*-Means Class-Labeled SVM

Baseline data (no stress), cage change (as routine maintenance), and intracranial surgery (as burden, in SD rats 8 animals at 7 time bins/in BDIX rats 7 animals at 7 time bins) were randomly partitioned into a training and test set (70%/30%). All following analyses were performed using the R software [R Core Team (2019), R: A language and environment for statistical computing, R Foundation for Statistical Computing, Vienna, Austria]. A scree analysis, which was run with the training set, revealed three classes as an optimum number of clusters for both strains. The blinded training set was class-labeled with unsupervised *k*-means clustering. Following cluster analysis, an SVM with a radial basis kernel was trained on the class-labeled training data using 5-times repeated 10-fold cross-validation and hyperparameter tuning. The decision boundaries are shown as a “map” of different shades of gray. The SVM was validated using the test data.

**FIGURE 4:**
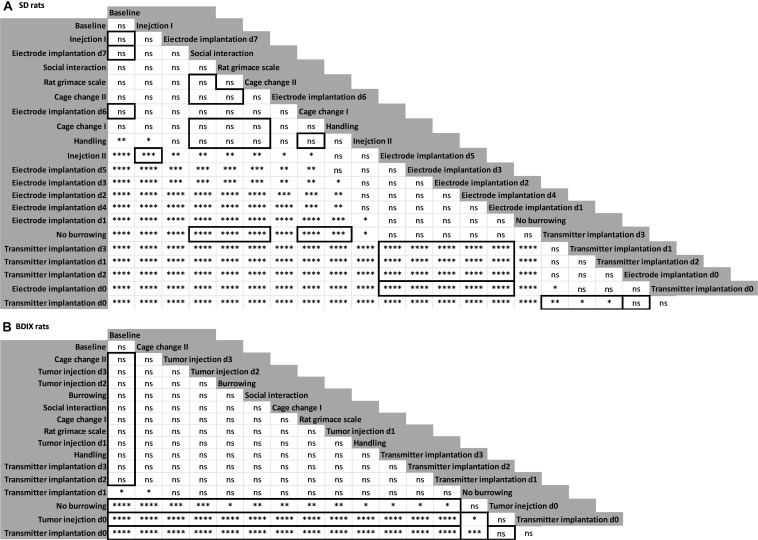
Statistical comparison of severity fractions of procedures. The fractions of data allocated to class high HR/low Act were statistically compared using a Fisher’s exact test; for SD rats **(A)** and BDIX rats **(B)**; framed fields that are described in the section “Results”; *P < 0.05, **P < 0.01, and ***P < 0.001.

To validate this model, the remaining data, measured after other procedures, were predicted using the SVM model. The fractions of data points lying in the different classes were counted, and procedures were ranked according to the allocation to the high HR/low Act class (Figure 3). The fractions of data lying in class high HR/low Act were ranked and statistically compared using a Fisher’s exact test performed in R (Figure 4).

#### Statistical Analysis

Further statistical analysis was performed using GraphPad Prism^®^ (v5, GraphPad Software, Inc., La Jolla, CA, United States). Values shown in Figure 1 are mean + standard error of the mean. In the remaining figures, data are shown for each animal. All data used in Figure 1 were analyzed to be normally distributed using the Shapiro–Wilk test, followed by Bartlett’s test to check for homoscedasticity, which revealed unequal variances (*P* < 0.05). Consequently, for statistical analysis, a non-parametric one-way repeated measure analysis of variance (one-way RM ANOVA, “Friedman test”) was carried out and in case of significance followed by Dunn’s multiple comparisons to baseline as a *post hoc* test. *P* < 0.05 was considered significant.

## Results

All rats well recovered from surgery without weight loss or deteriorated clinical conditions. Histology of BDIX rat brains after the study confirmed tumor growth in the prefrontal cortex of all rats. As the brains of SD rats were in total removed for molecular analysis, the location of the implanted electrodes was not analyzed. Nevertheless, previous studies showed correct placement of electrodes in about 90% of rats after stereotaxic implantation.

### Monitoring of Heart Rates and Changes in Activity During Experimental Procedures

As outlined in the experimental setup (Figures 1A,D), HR and Act were monitored after transmitter implantation, after distinct experimental procedures, and after intracranial surgery in SD and BDIX rats. Clinical score and body weight were not affected by either procedure (data not shown). In SD rats, statistical analysis of HR with ANOVA showed elevated measures after subcutaneous transmitter implantation (d0–d3: χ^2^ = 106.2, *P* < 0.0001, *post hoc P* < 0.001), after the day of intracranial electrode implantation (d0: χ^2^ = 106.2, *P* < 0.0001, *post hoc P* < 0.001), as well as after burrowing (χ^2^ = 106.2, *P* < 0.0001, *post hoc P* < 0.01; Figure 1B). Analysis of Act ANOVA only showed enhanced measures after burrowing (χ^2^ = 102.8, *P* < 0.0001, *post hoc P* < 0.01; Figure 1C).

For BDIX rats, analysis of different procedures with ANOVA revealed enhanced measures on the day of transmitter implantation and the first postoperative day (d0–d1: d0–d1: χ^2^ = 80.07, *P* < 0.0001, *post hoc P* < 0.05), as well as on the day of intracranial tumor cell injection (d0; d0: χ^2^ = 80.07, *P* < 0.0001, *post hoc P* < 0.01; Figure 1E). Analysis of Act showed enhanced measure for the day after subcutaneous transmitter implantation, as well as for the first cage changes analyzed (χ^2^ = 58.35, *P* < 0.0001, *post hoc P* < 0.001) and burrowing (χ^2^ = 58.35, *P* < 0.0001, *post hoc P* < 0.001; Figure 1F).

### Demarcation of Individual Time-Dependent Stress Reactions by *k*-Means Class-Labeled Machine Learning

Scatter plots of HR and Act with data during the first 4 h after baseline measurements, after cage change, and at the day of intracranial electrode implantation revealed stable HR and Act values during baseline and increased values up to 60 min after cage change. In contrast, after electrode implantation, HR was increased for more than 240 min but without increased Act (exemplarily for SD rats, see Figures 2A–C; BDIX rats, see Supplementary Figure 1).

For further analysis of a specific severity-related HR and Act pattern, a machine learning algorithm was applied. An SVM with radial basis kernel detected three classes within pooled and blinded data from baseline, after cage change, and after electrode implantation (exemplarily shown for SD rats in Figure 2D; for BDIX rats, see Supplementary Figure 1D). The respective classes are characterized by low HR/low Act (light gray), high HR/high Act (midgray), and high HR/low Act (dark gray).

Highlighting the training data sets (Figures 2A’–C’) from SD rats within the SVM model showed that baseline data are located in the low HR/low Act class, whereas the majority of data points after electrode implantation are allocated to the high HR/low Act class. The cage change data, however, were initially located in the high HR/high Act class, but returned to baseline values (low HR/low Act class) within the 4 h period.

The accuracy of the SVM for the training as well as the test set was 100% in both strains, with a 95% confidence interval of the training set (0.9315, 1) and test set (0.9687, 1) in SD rats [BDIX rats: training set (0.9229, 1); test set (0.9641, 1)].

### Classification of Experimental Procedures Using the SVM Classification Model

Next, the applicability of the trained SVM as a tool for assessing the impact of different procedures on the animals, thereby classifying experiments based on their severity, was tested. Therefore, the SVM model was applied to validation data sets of the respective procedures (four procedures are exemplarily shown in Figures 3A–D for SD rats and Figures 3E–H for BDIX rats). The percentage of data allocations into the three classes enabled a ranking of the procedures according to the fractions within high HR/low Act class (Figure 3I for SD rats and Figure 3J for BDIX rats).

In SD rats, the first days after surgical procedures, especially after transmitter implantation, were ranked the highest (Figure 3I). The day of surgery (d0) of transmitter implantation had the most counts in the high HR/low Act class (100%), followed by electrode implantation d0 (98.21%) with no significant difference (Figure 4A; *P* > 0.05). The days after both surgeries (starting at d1) displayed significantly lower counts in the high HR/low Act class than d0 (*P* < 0.05). The postsurgical days after electrode implantation had significantly lower counts in the high HR/low Act class than the postsurgical days after transmitter implantation (*P* < 0.001). Days 6 and 7 after electrode implantation did not show significant differences to baseline, anymore (*P* > 0.05).

Data points of SD rats, which did not burrow, ranked higher than those of electrode implantation on d1, although this comparison did not reach the level of significant difference, whereas in comparison to all other tested experimental procedures, it was significant (*P* < 0.001). The other experimental procedures showed only a few allocations to the high HR/low Act class and did not significantly differ from each other (*P* > 0.05).

In SD rats, Carprofen was injected subcutaneously on days 1 and 2 after electrode implantation. Additionally, antibiotics were administered for 8 days starting at 2 days before intracranial surgery. Data after the first presurgical subcutaneous injection of antibiotics (in Figure 3I, “Injection I”) ranked higher than the following injection (*P* < 0.001). Interestingly, this second presurgical injection was not significantly different from baseline measurements.

In BDIX rats, the day of transmitter implantation ranked at the top, followed by the day of intracranial tumor injection with no significant difference (Figure 3J). Data of BDIX rats that did not burrow ranked significantly lower than those of the surgical days, but higher than all other days and experimental procedures (*P* < 0.05). The postsurgical days of both surgeries (starting at d1), as well as the experimental procedures, were, except for the first day after transmitter implantation, not significantly different from baseline.

## Discussion

In animal experimentation, laws and guidelines require the assessment of the actual severity experienced by an animal. Evidence-based and validated methods and practical guidance for this purpose are still missing (Bleich and Tolba, 2017). Recently, computational methods have been introduced for severity assessment in this context (Häger et al., 2018; Möller et al., 2018; Abdelrahman et al., 2019).

In the present study, we showed that *k*-means labeling of HR and Act data measured in the home cage at baseline, after cage change, and after intracranial surgery with subsequent SVM-based machine learning approach computed three classes of conditions in the context of severity: one with low HR/low Act (baseline), one with high HR/high Act (exploratory “activity” after cage change), and one with high HR/low Act (“burden”-related intracranial surgery). Subsequent allocation of HR and Act data of experimental procedures of different domains into these classes allowed ranking of experimental procedures. Data of most behavioral and maintenance procedures were allocated in the low HR/low Act and high HR/high Act classes, as expected during resting condition and exploratory or stress-like behavior (Galani et al., 2001; Van Den Buuse et al., 2001; Sharp et al., 2002), whereas after different surgical procedures, data were initially allocated to the high HR/low Act class, as expected during burdensome procedures (Kramer et al., 1993; Arras et al., 2007; Greene et al., 2007; Wright-Williams et al., 2007; National Academy of Sciences US, 2009; Bourque et al., 2010; Cesarovic et al., 2011; Kumstel et al., 2020). This indicates that *k*-means labeled SVM may be useful for the assessment and grading of scientific procedures of different domains. More detailed analysis, however, not only highlighted differences between surgical procedures or repeated injections but also revealed unexpected allocations of behavioral procedures, such as “burrowing” into the class of “burden,” which may be used as a basis for refinement of experimental procedures when working with rats.

Postoperative data measured directly after surgery primarily showed counts in the high HR/low Act class with no differences between intracranial surgery and subcutaneous transmitter implantation in both rat strains. This is likely pain-related since enhanced HR and reduced Act have been reported after painful procedures (Kramer et al., 1993; Arras et al., 2007; Greene et al., 2007; Wright-Williams et al., 2007; National Academy of Sciences US, 2009; Bourque et al., 2010; Cesarovic et al., 2011; Kumstel et al., 2020), but may, at least in part, also be due to side effects of the general anesthesia.

Interestingly, depending on the respective intervention, the recovery time varied. The SVM analysis revealed that SD and BDIX rats seemed to be more and longer affected by subcutaneous transmitter implantation than by intracranial surgery for electrode implantation or tumor cell injection. One aspect may be that the brain itself has no pain receptors (Monitto et al., 2011), and that the surgery does not affect mobile parts of the body. Intracranial surgery may, therefore, be less painful than the surgery on the abdomen after subcutaneous transmitter implantation. With that regard, implantation of electrodes for deep brain stimulation in human patients is usually performed in the awake patient only with local anesthesia, whereas subsequent implantation of the pacemaker, which involves subcutaneous tunneling of the electrode’s lead to the subclavicular region, is performed under general anesthesia (Chen et al., 2017).

Data analysis using the SVM model also enabled severity grading of intracranial surgeries with different complexity. From the first postoperative day on, data measured after intracranial injection of tumor cells through a cranial burr hole in BDIX rats did not differ from those of baseline and different behavioral and handling procedures. In contrast, after intracranial electrode implantation in SD rats, which requires several screws wound to the skull as reinforcement for a head stage, data analysis showed allocations toward the high HR/low Act class (burden) until Day 5 after intracranial surgery when compared with data from baseline and handling procedures.

Pain medication was only given on the first two postoperative days, but the allocation of measures to the high HR/low Act class did not differ from those without pain medication. This suggests that the Carprofen regime is not sufficient for pain relief after subcutaneous transmitter implantation and intracranial implantation of electrodes. Strain differences with regard to pain sensitivity may also play a role, since after subcutaneous transmitter implantation BDIX rats seem to be less affected by surgery than SD rats. Interestingly, clinical score and body weight were not affected by either surgery, indicating that the clustering of HR and Act with the *k*-means algorithm is highly sensitive. This method may allow detecting even slight deviations from normal conditions that a flight animal like the rat does not show with an observer close by.

Another important finding was that no cumulative effect of repeated injections could be detected. While the initial data of the first subcutaneous injection of antibiotics before surgery were allocated to the high HR/low Act class, measures after the second injection did not differ from that after baseline recordings. Nevertheless, subcutaneous injections of antibiotics for 5 days after electrode implantation may aggravate postoperative measures, since these days are classified as more severe than the following 2 days without injection. So far, only a few studies have reported on the effect of repeated injections. Using voluntary wheel-running to assess severity in mice, no negative effect of repeated injections was found (unpublished observations). Nevertheless, negative effects were shown when mice were injected three times per day (Jirkof et al., 2015). With that regard, rats may better cope with handling- and distress-related procedures as mice.

Except for burrowing, the impact of all handling and behavioral procedures used for severity assessment did not substantially differ from routine animal handling for maintenance. Most measures are initially located in the high HR/high Act class, as expected with procedures that trigger exploratory behavior or as a reaction to unusual events, but within the next 4 h gradually returned to the low HR/low Act class that represents baseline condition. Importantly, even the reaction to temporary restraint in enclosures for the recording of the Rat Grimace Scale (Langford et al., 2010; Sotocinal et al., 2011) did not differ from maintenance and handling procedures. With that regard, an increased HR is a typical response to stressors by an organism and does not necessarily harm an animal. Accordingly, it has previously been described as a stress-like response to cage change and other experimental procedures (Sharp et al., 2002).

The only exception in the experimental procedures tested here was the reaction to a burrowing tube placed into an empty cage that—as species-specific behavior—has recently been proposed to be useful for severity assessment in rats (Seiffert et al., 2019). When first exposed to the burrowing tube, all SD rats and almost all BDIX rats did not engage in burrowing at all. In these non-burrowing rats, telemetric measurements were allocated mostly in the high HR/low Act class. While postoperative allocation to the high HR/low Act class likely indicates pain, or distress-related states, when placed in the burrowing setup, measures in this class rather indicate a strong burden related to anxiety and discomfort in a foreign environment. This corroborates a previous study showing that in response to anxiety, HR was elevated, and Act was decreased (Zhang et al., 2004). Interestingly, data of rats that immediately started burrowing upon first exposure to the burrowing tube mainly resulted in an Act-related increase of HR when back in the home cage, similar to other behavioral procedures that triggered exploratory behavior. Furthermore, rats that were raised in highly enriched large cages, including repeated exposure to foreign and potentially threatening equipment, showed better performance in the initial phases of behavioral testing for cognitive function (Hoffmann et al., 2009). Our observation may, therefore, trigger the discussion of whether rats that will be exposed to behavioral testing should be housed in highly enriched cages to get used to new and potentially threatening behavioral setups.

The main limitation of this work is that so far, classification is based on eight SD and seven BDIX rats. Further recordings will likely result in more general and robust *k*-means clustering and more specific classification of different procedures. It also remains open to what extent age and sex will have an impact on classification since in the present study, only male rats with roughly the same age and weight have been used. In addition, because of technical constraints, all recordings were taken immediately *after* the actual procedure. Future studies should therefore also include measures during the actual procedure. Furthermore, the attribution of data to the high HR/low Act class only indicates deviation from the normal state of the animal. Whether this is related to pain (as likely explanation after surgery) or anxiety (as likely during testing in foreign behavioral setups) needs to be tested with more specific methods. Finally, since subcutaneous transmitter implantation is invasive itself and had more impact than intracranial surgery, this setup should only be used for grading of certain key animal models and experimental procedures and not as routine severity assessment method.

We are aware that using different neurosurgical procedures may be a limitation of the current study. Apart from intracranial surgery, however, both rat strains were subjected to the same procedures (transmitter implantation, maintenance procedures, and behavioral testing), which enable direct comparison of reactions between strains. In all procedures, BDIX rats were less affected than SD rats. The better outcome after intracranial injection of tumor cells in BDIX rats may therefore be related not only to the less severe surgery but also to the constitution of the BDIX strain. Differences of the autonomic system between strains may at least in part account for this different susceptibility to the different procedures. Accordingly, the algorithm defined clusters for each rat strain results in slightly different demarcation of clusters. Nevertheless, the allocation and ranking of procedures to the clusters in both strains were relatively similar and therefore allowed comparisons between strains.

Together, we here describe that SVM machine learning of HR and Act measured in the home cage may be useful for evidence-based severity assessment and ranking of experimental procedures of different domains, which is legally required, essential for a realistic harm/benefit analysis and development of refinement strategies. Furthermore, our model detects not only postoperative pain but also anxiety- and stress-related responses and thus provides a tool for the detection of multidimensional burden.

## Data Availability Statement

The original contributions presented in the study are included in the article/Supplementary Material, further inquiries can be directed to the corresponding author/s.

## Ethics Statement

The animal study was reviewed and approved by the Lower Saxony State Office for Consumer Protection and Food Safety (LAVES, license 18/2837).

## Author Contributions

LW, KS, and CH wrote the manuscript. LW, SH, and A-KR conducted the experiments. ST conducted the statistical analysis. All authors edited and reviewed the manuscript.

## Conflict of Interest

The authors declare that the research was conducted in the absence of any commercial or financial relationships that could be construed as a potential conflict of interest.

## Supplementary Material

Supplementary Figure 1 Development of classification model in BDIX rats. Heart rate (HR; bpm) and activity (Act; counts/min) until 4 h after experimental procedures of baseline as no stress **(A)**, cage change as stress-like response inducing procedure **(B)**, and tumor injection as distress **(C)**. **(A–C)** served as training data set for development of SVM decision boundaries map **(D)** with blinded training data set (black symbols). SVM identified three classes: dark gray: high HR/low Act, midgray: high HR/high Act, light gray: low HR/low Act. Overlay of training data decision boundaries **(A’–C’)**. HR is shown as mean, and Act is shown as sum over 10 min starting at the indicated time points (color coded). Symbols indicate different animals.

Supplementary Figure 2 Scree-plot and *k*-means. Results of scree-plot analysis for SD **(A)** and BDIX rats **(C)** as well as the results from the *k*-means class-labeling of the blinded data for SD **(B)** and BDIX rats **(D)**. Colors indicate cluster allocations of data by *k*-means.

## Abbreviations

AB: s.c. injection of antibiotics
Act: activity
BDIX: BDIX/UImHanZtm rats
bpm: beats per minute
BSL: baseline
BUR: burrowing
Ca: Carprofen
CG: cage change
d: day
EI: intracranial electrode implantation
Handl: handling
HR: heart rate
i.p.: intraperitoneal injection
Inj.: subcutaneous injection
min: minute
No BUR: animals that did not burrow
RGS: Rat Grimace Scale
s.c.: subcutaneous injection
SD: Crl:CD(SD) rats
SI: social interaction
SVM: Support Vector Machine
TI: transmitter implantation
TuIn: intracranial tumor injection.
