# Supplementary figures and images for: Monitoring of Heart Rate and Activity Using Telemetry Allows Grading of Experimental Procedures Used in Neuroscientific Rat Models
Source: Front Neurosci. 2020 Dec 17;14:587760. doi: 10.3389/fnins.2020.587760 (PMC7793729; doi:10.3389/fnins.2020.587760)

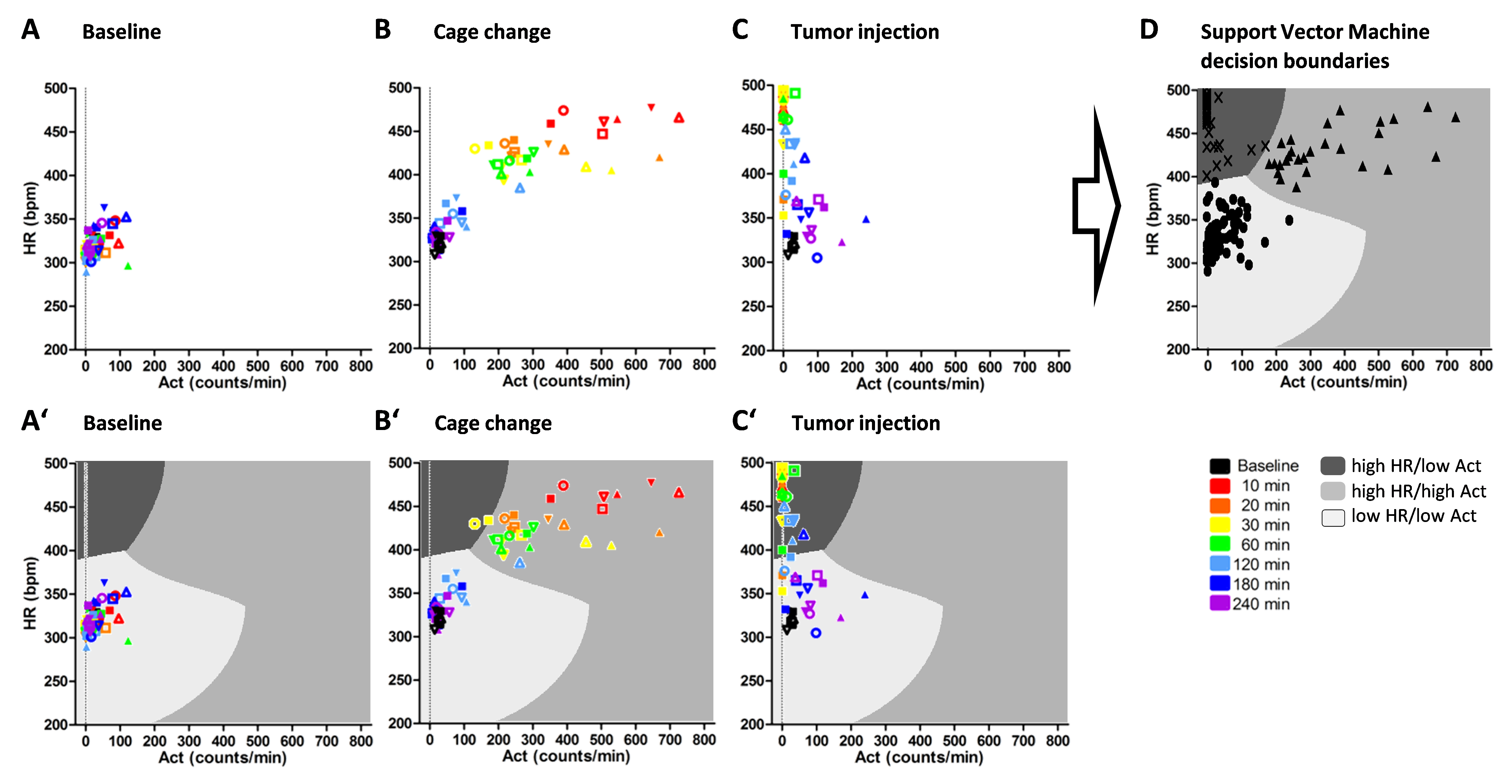

Supplement: Supplementary Figure 1 — Development of classification model in BDIX rats. Heart rate (HR; bpm) and activity (Act; counts/min) until 4 h after experimental procedures of baseline as no stress (A), cage change as stress-like response inducing procedure (B), and tumor injection as distress (C). (A–C) served as training data set for development of SVM decision boundaries map (D) with blinded training data set (black symbols). SVM identified three classes: dark gray: high HR/low Act, midgray: high HR/high Act, light gray: low HR/low Act. Overlay of training data decision boundaries (A’–C’). HR is shown as mean, and Act is shown as sum over 10 min starting at the indicated time points (color coded). Symbols indicate different animals. [file Image_1.TIF]

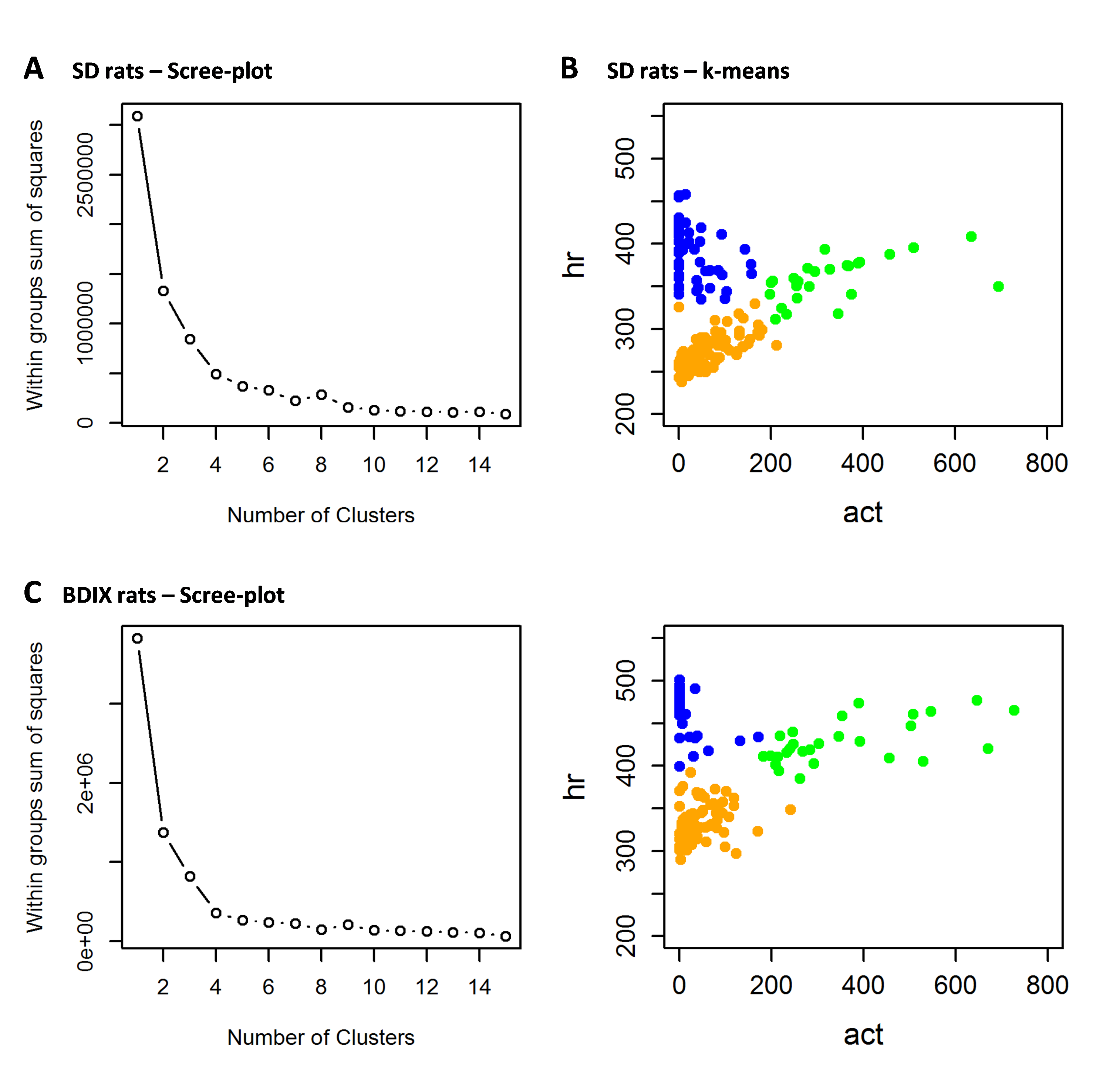

Supplement: Supplementary Figure 2 — Scree-plot and k-means. Results of scree-plot analysis for SD (A) and BDIX rats (C) as well as the results from the k-means class-labeling of the blinded data for SD (B) and BDIX rats (D). Colors indicate cluster allocations of data by k-means. [file Image_2.TIF]
